# Sterilization of Biofilm on a Titanium Surface Using a Combination of Nonthermal Plasma and Chlorhexidine Digluconate

**DOI:** 10.1155/2017/6085741

**Published:** 2017-09-19

**Authors:** Tripti Thapa Gupta, Surya B. Karki, Jyl S. Matson, Daniel J. Gehling, Halim Ayan

**Affiliations:** ^1^Department of Bioengineering, College of Engineering, The University of Toledo, Toledo, OH 43606, USA; ^2^Department of Medical Microbiology and Immunology, The University of Toledo College of Medicine and Life Sciences, Toledo, OH 43614, USA; ^3^Department of Orthopedic Surgery, The University of Toledo Medical Center, Toledo, OH 43614, USA; ^4^Department of Mechanical, Industrial, and Manufacturing Engineering, College of Engineering, University of Toledo, Toledo, OH 43606, USA

## Abstract

Nosocomial infections caused by opportunistic bacteria pose major healthcare problem worldwide. Out of the many microorganisms responsible for such infections,* Pseudomonas aeruginosa* is a ubiquitous bacterium that accounts for 10–20% of hospital-acquired infections. These infections have mortality rates ranging from 18 to 60% and the cost of treatment ranges from $20,000 to $80,000 per infection. The formation of biofilms on medical devices and implants is responsible for the majority of those infections. Only limited progress has been made to prevent this issue in a safe and cost-effective manner. To address this, we propose employing jet plasma to break down and inactivate biofilms* in vitro*. Moreover, to improve the antimicrobial effect on the biofilm, a treatment method using a combination of jet plasma and a biocide known as chlorhexidine (CHX) digluconate was investigated. We found that complete sterilization of* P. aeruginosa* biofilms can be achieved after combinatorial treatment using plasma and CHX. A decrease in biofilm viability was also observed using confocal laser scanning electron microscopy (CLSM). This treatment method sterilized biofilm-contaminated surfaces in a short treatment time, indicating it to be a potential tool for the removal of biofilms present on medical devices and implants.

## 1. Introduction

Adherence of bacteria to implanted medical devices and damaged tissues can lead to biomaterial-associated infections (BAIs), often resulting in severe disease and implant failures. BAI is largely due to the ability of the bacteria to encase and protect themselves in a matrix composed of polysaccharide and protein, known as a biofilm [1, 2]. Initial bacterial colonization of a foreign implanted material usually occurs either by direct inoculation or by hematogenous spread. Later, when the organisms form biofilms, neither antibiotics nor immediate surgical debridement is effective in removing them from surfaces. Instead of direct debridement, removal of the medical device or internal prosthesis is the primary surgical treatment for such BAIs. However, removal is associated with increased patient morbidity and mortality, and it causes higher healthcare cost because of repeated surgeries, extended hospitalization, rehabilitation, and antibiotic therapy. For example, a single prosthetic joint BAI has an average, estimated healthcare related cost of at least $50,000 and up to $130,000 [3–5]. Furthermore, these costs underestimate the true impact to the patient, as this cost does not include the long-term physical and social impairments that the patients potentially endure [6, 7].

Typical BAIs in the healthcare setting include those on dental implants, prosthetic joint replacements, catheters, cardiac pacemakers, and heart valves [8–12]. In some cases such as catheters, treatment by simple removal of the device causes little harm to the patient. In other cases, such as prosthetic joint replacements, removal is very invasive and has the potential to be an unfavorable experience for a patient. Therefore, new alternatives in the treatment of BAIs would be beneficial in certain clinical circumstances.

Use of nonthermal plasma in combination with CHX could conceivably be a suitable antimicrobial tool for removing these biofilms in clinical settings; however, very limited information has been published on this research topic. We therefore set out to study the antimicrobial effect of jet plasma and CHX on a surface colonized with a biofilm. Out of the many bacteria capable of forming biofilms,* P. aeruginosa* is well known for its severe adverse effects on medical implants and is responsible for about 75% of biofilms found on medical implants and devices [12]. Hence, the primary aim of this research study is to investigate the possibility of sterilizing* P. aeruginosa* biofilms using a combination of plasma and CHX.

Plasma is one of the four fundamental states of matter, with the others being solid, liquid, and gas. Plasma is a cocktail of positively and negatively charged ions, electrons, neutral atoms, and molecules [13]. It has been extensively used in many research areas, including sterilization of implant surfaces, surface modification [14],* in vitro* blood coagulation [15], wound healing and disinfection [16, 17] and tissue regeneration and in the treatment of various infections [18], bacterial decontamination and sterilization [19], dental cavities [20], and various cancers treatment [21]. The efficacy of plasma treatment could be increased by modulating several factors, such as plasma treatment time [22], frequency [23], electrical input power [24], and addition of different gases such as helium, argon, nitrogen [25], and oxygen [26] as shown in other studies. However, in order to implement the plasma treatment methodology in a clinical setting in the future, it needs to be both safe and effective. Thus, in this study, plasma was applied in conjunction with commonly used biocide known as chlorhexidine (CHX) digluconate with clinically safe doses to explore their combinatorial antimicrobial efficacy.

Chlorhexidine (CHX) is one of the most widely used antiseptics for decontaminating skin, oral, and medical devices. In healthcare usage, chlorhexidine digluconate is one of the common forms of chlorhexidine. It has various clinical applications, being used as a topical antiseptic on the skin and as an oral medicine for preventing dental plaques. Moreover, chlorhexidine is often impregnated into dental implants, vascular catheters, IV connectors, and dressings to avoid bacterial colonization and biofilm formation [27]. The positively charged chlorhexidine molecule binds with the negatively charged phosphate groups on the bacterial cell wall, thereby altering the cells osmotic equilibrium [28]. This leads to an increase in cell wall permeability that allows CHX molecules to enter into the bacterial cell, causing leakage of cytoplasmic contents and cell death [29]. CHX has both bacteriostatic and bactericidal effects when used at low and high concentrations, respectively [30]. The bactericidal effect is due to precipitation and/or coagulation of bacterial cytoplasmic contents caused by protein cross-linking [29]. The bacteriostatic effect of CHX is due to leakage of phosphorus and potassium from the cell [29, 31]. 2% CHX has been commercially used as an oral rinse with no adverse effects [30]. Several studies have examined varying CHX concentrations ranging from 0.1% to 2% and verified them to be toxicologically safe [30].

Since neither plasma nor CHX at clinically safe doses is completely effective at eradicating BAIs, the key objective of the present study is to determine the efficacy of a combination treatment approach using plasma and CHX for the reduction of bacterial biofilms compared to using plasma and CHX treatment individually. We hypothesized that adding CHX to plasma treatment might increase the antimicrobial efficacy over that of CHX or plasma treatment alone. Moreover, we hypothesized that the biofilm might be better destroyed by plasma treatment first, allowing CHX to penetrate into the bacterial cell wall more easily, causing additional cell damage.

## 2. Materials and Methods

### 2.1. Bacterial Strain and Culture Conditions

*Pseudomonas aeruginosa* PAO1 (ATCC® BAA 47™, Manassas, VA, USA) was stored and cultured in tryptic soy broth (TSB) (Thermo Fisher Scientific, Hanover Park, IL, USA). Bacterial stocks were maintained in 20% glycerol at −80°C.

### 2.2. Biofilm Growth on a CDC Biofilm Reactor

Biofilms were grown on a titanium coupon (diameter of 12.7 mm and thickness of 3 mm) on a Center for Disease Control and Prevention (CDC) biofilm bioreactor (BioSurface Technologies, Bozeman, MT, USA) for 24 hours in batch phase and then 24 hours under dynamic phase with agitation. Tryptic soy broth (TSB) was used as the media for growing biofilms in the reactor at 37°C for 48 hours. An overnight culture of PAO1 was adjusted to an optical density (OD600) equivalent to 10^8^ CFU/ml. The standardized bacterial suspension was used to inoculate the reactor. For the entire 48 hours, shear stress was produced by the baffle of biofilm reactor rotating at a speed of 130 rpm to avoid the presence of planktonic bacteria. After the selected growth time, the coupons were aseptically removed from the reactor and subjected to combinatorial treatment with jet plasma and CHX for various exposure times under sterile conditions. Treatment with 0.9% NaCl was used as negative control. CHX and plasma treatment individually were both used as positive controls. After treatment, treated and control biofilms were suspended in a tube with Phosphate-Buffered Saline (PBS) and sonicated for 5 minutes in an ultrasonic bath (CPX2800H, Branson Ultrasonics Corp., Danbury, CT, USA) with vortexing for 30 seconds. The bacterial suspension was serially diluted and plated in triplicate on TSB agar. Plates were incubated at 37°C for 24 hours and colonies were counted.

### 2.3. Jet Plasma Generation

**Figure 1:**
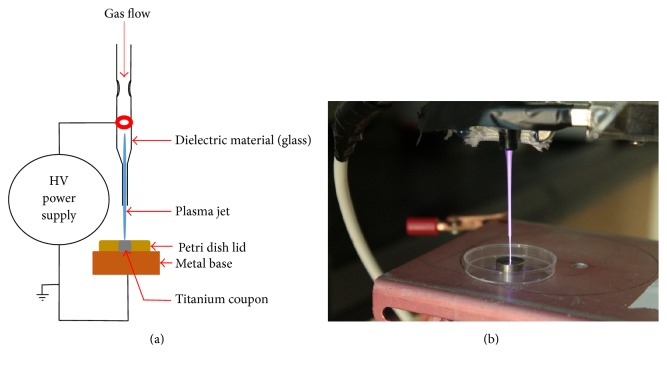
Schematic diagram and photograph of the jet plasma setup. (a) demonstrates the schematic diagram of the jet plasma and (b) shows the actual experimental setup of jet plasma.

A schematic diagram and a photograph of the jet plasma used in this study are shown in Figure 1. The plasma operates at 1 kHz frequency with 10 kV. It consists of a quartz tube with an inner diameter of 1 mm. A copper electrode (diameter of 1.19 mm) encircles the tube. The distance between the jet nozzles and sample is maintained at 10 mm. The plasma jet was operated with a gas mixture of 100% helium (He) gas at a total flow rate of 1 Standard Liters per Minute (SLPM) into ambient air. The flow rate was controlled by a flow controller (FMA-1607A, Omega Engineering Inc., Norwalk, CT, USA). Helium gas was chosen for its inertness and homogenous generation of nonthermal plasma. The temperature on the titanium coupon was measured with an infrared thermometer (NUB8380H, Nubee, Duarte, CA, USA). The temperature was noted to be 27°C during the 15 minutes of plasma treatment and remained the same after plasma treatment.

### 2.4. Electrical Characterization of Jet Plasma

**Figure 2:**
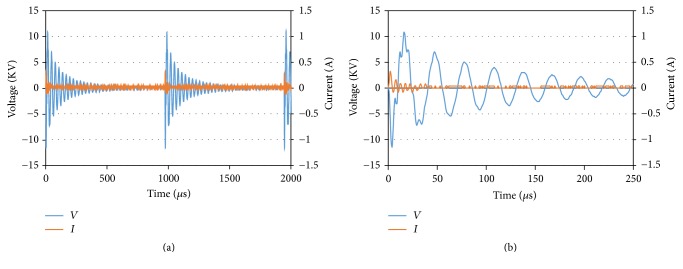
(a) Voltage and current waveforms of Jet plasma. Two complete cycles based on 1000 Hz frequency and 4 *µ*s pulse width are shown. (b) A close-up view of the voltage and the current waveforms.

High voltage and current waveforms of the jet plasma system were analyzed using a digital oscilloscope. A high voltage probe (1000 : 1) was connected in parallel with a plasma electrode and a high voltage cable was passed through a current probe (1 VA^−1^). By using the digital oscilloscope (TDS 2014C, Tektronix, Melrose, MA, USA), the changes in voltage and current waveforms over time were recorded (Figure 2).

### 2.5. Optical Emission Spectroscopy Analysis

Jet plasma combined with He gas was characterized using optical emission spectroscopy (OES) to detect the reactive species generated in the plasma. OES was performed using a Jaz fiber optic spectrometer (JAZA 2497, Ocean Optics, Dunedin, FL, USA). The spectrometer was calibrated using a standard mercury-argon light source (HG-1, Ocean Optics, Dunedin, FL, USA). The light source was connected to the spectrometer using a fiber optic cable (core diameter: 600 um, QP600-1-UV-VIS). After calibration, one end of the optical fiber was fixed in a position near the plasma glow and the emission spectrum was monitored. Data from the spectroscopy was transferred to the computer using Spectra Suite software (Ocean Optics, Dunedin, FL, USA) via USB. The integration time of the collected data was set to 100 ms. This spectroscopy system allows the user to observe the entire spectrum instantly while doing the spectroscopy procedure. Spectrum data was then exported to Microsoft Excel for further analysis.

### 2.6. CHX Treatment

Titanium coupons containing biofilms were treated with 1% CHX for the indicated time periods. A 1% solution of CHX was made in sterile DI water diluted from a 20% CHX stock solution. For treatment with CHX, titanium coupons containing biofilms were submerged in 700 *µ*l of CHX in a 24-well plate and incubated for the indicated periods of time. After treatment with CHX, the antiseptic effect was halted by adding 700 *µ*l of inactivating agent for the same time period that was used for CHX treatment. The CHX and all of the inactivation agents were purchased from Thermo Fisher Scientific (Norwalk, IL, USA). The inactivation agent consists of a solution of Tween 80 (30 g·l^−1^), Saponin (30 g·l^−1^), Histidine (1 g·l^−1^), and Cysteine (1 g·l^−1^) [32]. The amount of inactivators was proven by the quantitative suspension test according to DIN EN 1040 (German Institute for Standardization) [32]. We also determined whether the inactivation agent had any antimicrobial effect that would confound these experiments. The treatment was performed on planktonic bacteria and biofilms (Table 1), showing that the inactivation agent does not have antimicrobial activity. The treatment time with inactivation solution was 15 minutes.

### 2.7. Treatment Order Variation and Abbreviation

We tested whether plasma and CHX treatment order would affect biofilm disruption. The treatment methods hereafter are referred to as “C + P” (CHX + plasma) when the biofilm is treated first with CHX and second with plasma and “P + C” (plasma + CHX) when the biofilm is treated with plasma first and CHX second. The treatment times for both methods were 5, 10, and 15 minutes with C + P 5 minutes representing treatment with CHX for 5 minutes and plasma with 5 minutes and so on for 10 and 15 minutes as well.

### 2.8. Quantitative (Colony Count Method and XTT Assay) Assessment of Biofilms

After the biofilms have been treated, the viability of surviving cells was quantitatively determined using a standard colony count and XTT viability assay. For the colony count method, the bacterial suspension was serially diluted and plated on a TSB agar plate. After 24 hours of incubation, the number of colonies was counted manually.

The XTT assay quantifies the metabolically active cells as represented by the presence of an orange formazan product that results from XTT reduction by metabolically active cells. The presence of metabolically active or surviving cells results in an increase in absorbance at 450 nm. The absorbance value was recorded after treating biofilms as described in Results. The XTT viability assay (TOX2, Sigma-Aldrich, St. Louis, MO, USA) was carried out according to manufacturer's instructions. The XTT stock solution was prepared by reconstituting a kit vial (containing 5 mg of XTT with 1% PMS) with 5 ml of PBS. 20 *µ*l of the XTT stock solution was added to the 96-well plate containing 50 *µ*l bacterial suspension and 50 *µ*l media (TSB). The plate was then incubated overnight at 37°C. Absorbance at 450 nm was recorded after incubation to quantify the XTT metabolic product. Blank controls (50 *µ*l media, 50 *µ*l PBS, and 20 *µ*l XTT solution) were also included.

### 2.9. Qualitative Scanning Electron Microscopy (SEM) and Confocal Laser Scanning Microscopy (CLSM) Assessment of Biofilms

For SEM (Quanta 3D FEG, FEI, Hillsboro, OR, USA) images, the treated and the control titanium coupons containing biofilms were fixed according to the following procedure described elsewhere [33, 34] with some modifications. The chemicals used for SEM and CLSM were purchased from Thermo Fisher Scientific (Norwalk, IL, USA). All of the coupons were placed in 24-well plates and exposed to prefixing agents containing 2.5% Glutaraldehyde in 0.2 M cacodylate buffer (pH 7.4). The coupons were soaked with prefixing agents for 24 hours at room temperature. The coupons were then rinsed with cacodylate buffer three times for 5–10 minutes, placed in 2% osmium tetroxide solution in cacodylate buffer for 2 hours, and rinsed with cacodylate buffer three times for 5–10 minutes. After the final rinse, the coupons were dehydrated by placing in increasing concentrations of ethanol (70%, 90%, and 100%) three times each for 20 minutes. Finally, the coupons were dried with 100% Hexamethyldisilazane (HMDS) two times each for 20 minutes. The coupons were placed in a desiccator overnight, before being imaged using SEM after gold-coating for 40 seconds.

For CLSM (TCS SP5, Leica Microsystems, Buffalo Grove, IL, USA) images, the treated and the control titanium coupons containing biofilms were stained with the BacLight Bacterial Viability Assay (L7012, Thermo Fisher Scientific, Norwalk, IL, USA). For this, equal proportions of both dyes (SYTO9 and PI) were mixed with sterile DI water according to manufacturer's instructions to selectively stain live (green) and dead (red) cells. 1 ml of the mixed dye was added to the 24-well plate containing the coupons and incubated in the dark for 15 minutes. The coupons were then washed three times with PBS and postfixed with a 4% paraformaldehyde solution for 30 minutes. The fixed biofilm cells were further washed with PBS and subsequently immersed in PBS to view under CLSM.

## 3. Statistical Analysis

All experiments in this study were run in triplicate and each experiment was repeated at least three times. All of the statistical differences were determined using *t*-test in Microsoft Office Excel data analysis add-in with a 95% confidence interval (*P* < 0.05). A *P* value less than 0.05 was considered to be statistically significant. All values are reported as the mean ± standard deviation of the mean.

## 4. Results

### 4.1. Electrical Characterization of Jet Plasma

The voltage and current waveforms of jet plasma over time are shown in Figure 2. These kinds of waveforms are typically observed as a result of electrical characterization of nonthermal plasmas [35, 36]. A close-up view of an oscillogram with a few voltage peaks and current is presented in Figure 2(b). This kind of sinusoidal waves identifies the micro discharges ignited with 1000 Hz frequency and 4 *μ*s pulse width with the maximum applied high voltage of 10 kV.

### 4.2. Optical Emission Spectroscopy Analysis of Jet Plasma

**Figure 3:**
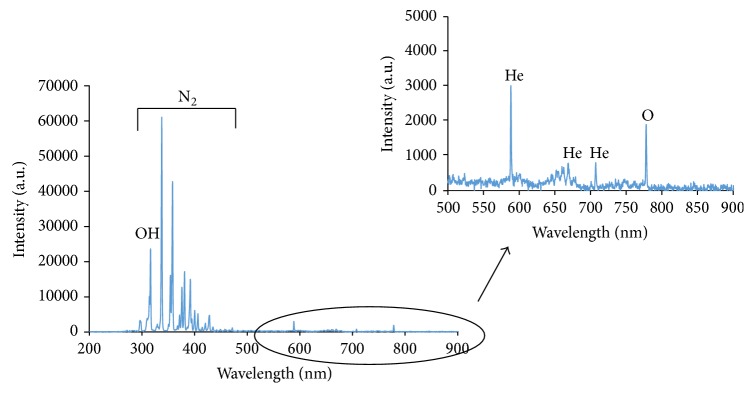
Typical emission spectrum of He plasma jet using 1000 Hz pulse frequency and 4 *µ*s pulse width (measured at output of 10 kV and He flow rate of 1 SLPM).

Figure 3 shows the emission spectra of the He plasma jet in the wavelength ranges from 200 nm to 900 nm. The OH molecular spectrum (~306–310 nm), N_2_ molecular spectrum (~330–425), excited atom emission lines (~777), and He lines were observed as a result of the helium plasma jets interacting with ambient air [37, 38]. Between 200 nm and 300 nm, weak emissions are detected, known as NO [39].

### 4.3. Jet Plasma Treatment of Biofilms Using a Combination of Plasma and CHX Treatment

**Figure 4:**
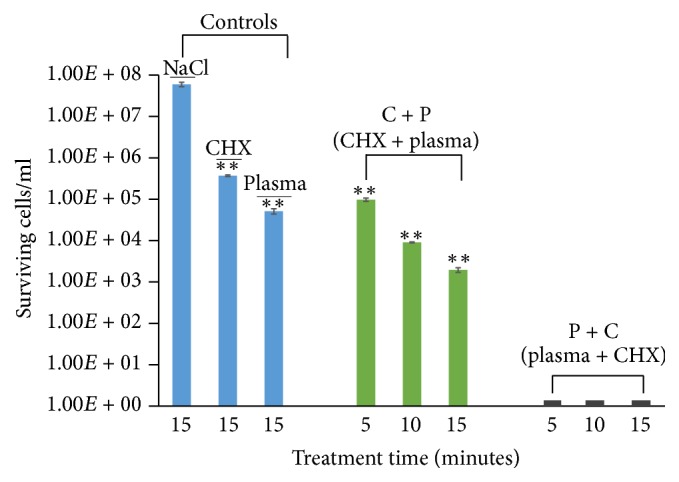
Surviving bacterial cells of control and treated (C + P and P + C) biofilm. Titanium coupons containing biofilms were treated for different time intervals at 1 kHz frequencies using He jet plasma and CHX. After treatment, the coupon was subjected to the colony count assay (as described in Materials and Methods) and the number of viable cells was calculated. The results are expressed as mean ± standard deviation of cell number (*n* = 3). Asterisks (*∗∗*) denote statistical significant differences between NaCl and other treatment groups (C + P, P + C, CHX only, and plasma only), respectively (*P* < 0.01). P + C (black bar) in the graph represents 0 cells/ml.

In this study, the combined antimicrobial effect of jet plasma and CHX was tested as a possible treatment to disrupt biofilms grown under dynamic flow conditions for a total of 48 hours. Out of the two treatment modalities (CHX first, C + P, or plasma first, P + C), P + C was able to completely sterilize a titanium surface containing a biofilm within 5 minutes as determined by colony count assay (Figure 4). Decontamination was also achieved after C + P treatment. The C + P method reduces the bacterial cells by 2.78 log (99.9%), 3.81 log (99.99%), and 4.48 log (~99.999%) at 5, 10, and 15 minutes of treatment, respectively, as shown in Figure 4. CHX treatment alone also reduces bacterial numbers (2.2 log, 99%) as done by plasma treatment alone (3.06 log, 99.9%) after 15 minutes. However, the combination treatments were significantly more effective than either treatment alone.

**Figure 5:**
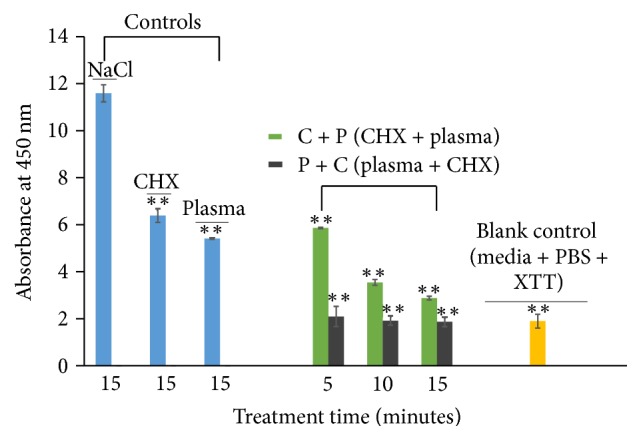
Metabolically active bacterial cells of control and treated (C + P and P + C) biofilms. Titanium coupons containing biofilms were treated for different time intervals at 1 kHz frequencies using He jet plasma and CHX. After treatment, the coupon was subjected to the XTT assay (as described in Materials and Methods) and the absorbance reading was measured using a spectrophotometer. The results are expressed as mean ± standard deviation of absorbance reading (*n* = 3). Asterisks (*∗∗*) denote statistical significant differences between NaCl and other treatment groups (C + P, P + C, CHX only, plasma only, and blank control), respectively (*P* < 0.01).

In addition to the colony count assay, we cross-validated the viability results via XTT assay which demonstrated similar outcomes [40]. The negative control NaCl in Figure 5 shows a higher absorbance value, which signifies the presence of viable cells. In contrast, the absorbance reading of P + C and C + P continuously drops with increasing plasma exposure time, which indicates the reduction in the number of metabolically active cells after treatment. Moreover, the absorbance value of the blank control (no biofilm) is similar to P + C treatment, which suggests the absence of metabolically active cells in the P + C treatment. However, some cells were still observed to be active or viable in the C + P treated biofilms and in the positive controls (CHX and plasma only) as shown by absorbance reading in Figure 5. Therefore, the above data from colony count and XTT assay demonstrate that biofilm cells can be killed using plasma and CHX (P + C) as a combined treatment methodology and that it is significantly more effective than treatment with each of them individually.

### 4.4. Scanning Electron Microscopy Reveals Disruption of the Biofilm Surface upon Combinatorial Treatment with Plasma and CHX

**Figure 6:**
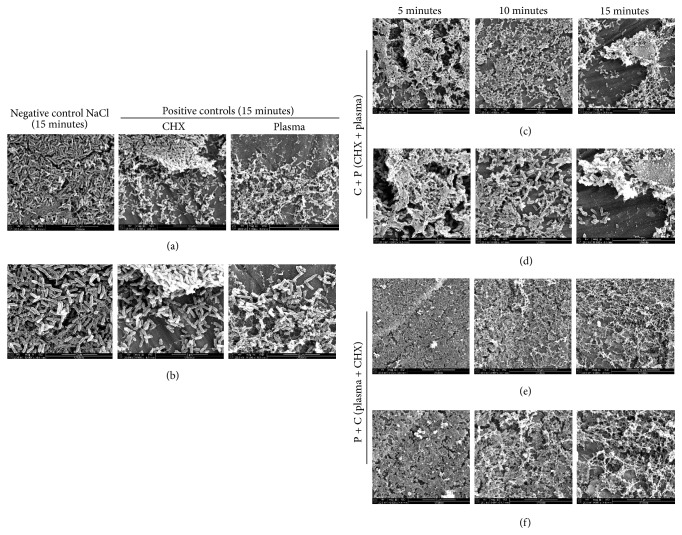
SEM images of control and treated (C + P and P + C) biofilms on titanium coupons. The titanium coupons (both treated and control) containing biofilms were subjected to SEM and representative images were taken from the coupon. (a) SEM images of biofilms of negative (NaCl) and positive controls (CHX and plasma only). (c, e) SEM images of biofilms after combinatorial treatments: (c) C + P and (e) P + C at different treatment times. Magnification is 5000x, 10 *µ*m, for (a), (c), and (e) and 10000x, 5 *µ*m, for (b), (d), and (f).

SEM images of the NaCl treated biofilms were visibly intact and rod-shaped (Figure 6). The biofilm treated with CHX alone for 15 minutes shows visible disruption of the 3D biofilm structure, with scattered cells and biofilm clumps observable on the titanium surface (Figure 6(a)). When biofilms were treated with plasma alone for 15 minutes, the micrograph images display bacteria embedded within the ECM with more biofilm degradation. Biofilm clumps were not visible in the sample treated with plasma (Figures 6(a) and 6(b)) only as seen on CHX treated sample alone; instead scattered cells were observed. The combination treatments, C + P and P + C, show more biofilm disruption in comparison to any of the individual treatments. Moreover, P + C treatment demonstrated more effectiveness on removing biofilm than C + P treatment. With C + P treatment, along with biofilm degradation, the images demonstrated biofilm clumps and scattered individual bacterial cells over the titanium surface along with some biofilm clumps encased within the matrix. However, in P + C treatment, more biofilm residue or remnants were encountered with very few intact bacteria (Figure 6(f)) on the titanium surface. This again shows that there was more biofilm destruction from the P + C treatment than when the combination treatment was performed in the opposite order.

### 4.5. Live and Dead Biofilm Cells Observed under Confocal Microscope

**Figure 7:**
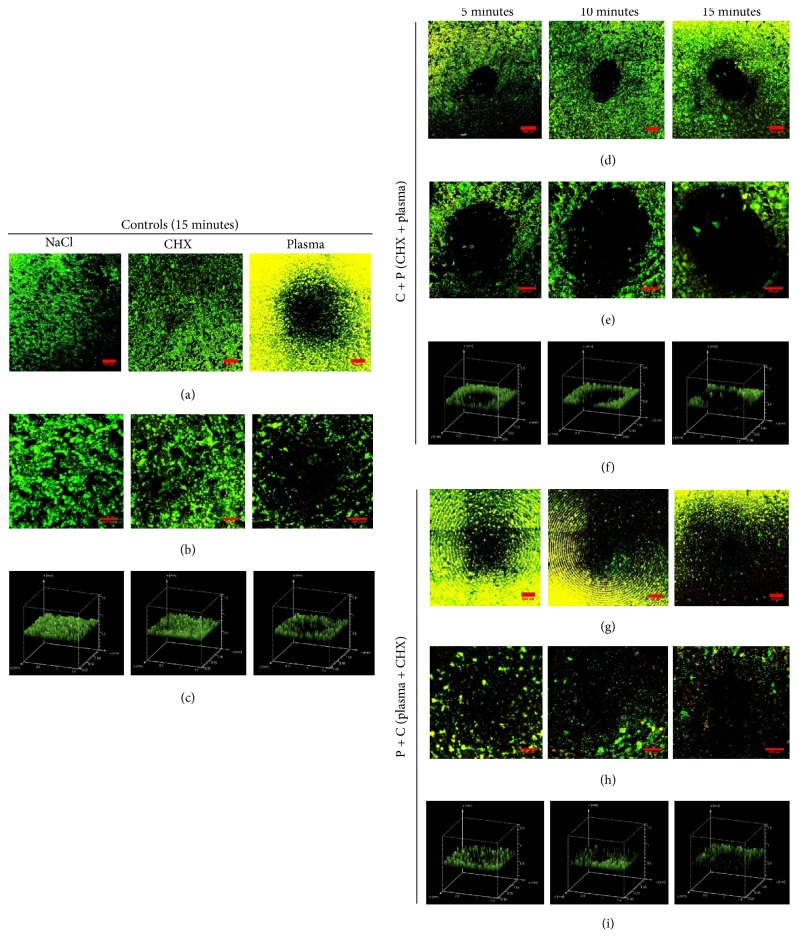
Confocal laser scanning microscope (CLSM) images of biofilms at titanium coupons in controls and combination treatment group: C + P and P + C. The titanium coupons (both treated and control) containing biofilms were subjected to CLSM and representative images were taken from the coupon. (b), (e), and (h) show 2D confocal images of control and treated (C + P and P + C) biofilms and (c), (f), and (i) represent the 3D volume image of the control and treated (C + P and P + C) biofilms, respectively. (a), (d), and (g) are merged images with multiple images. (b), (e), and (h) are a single image at the treated region. Green coloration and yellow coloration represent live and dead cells as stained by SYTO9 and PI (propidium iodide), respectively. The round black region at the center is the area directly treated by the plasma jet. The scale bar is 250 *µ*m.

To further determine the antimicrobial effect of the two different treatment methods visually, we stained the biofilms with SYTO9 and PI. Using CLSM, the difference between the controls and the treated biofilms (C + P and P + C) was observed. The negative NaCl control biofilm shows the intact viable cells represented by green coloration in the microscopic images (Figures 7(a)–7(c)). The biofilms were also intact after CHX treatment (Figures 7(a)–7(c)). Compared to NaCl control, fewer cells appear to be viable as indicated by yellow colored (dead) cells in the plasma only treatment (Figures 7(a)–7(c)). In all of the images where plasma has been applied, a circle is seen corresponding to the area treated directly by the plasma tip. We can see more dead cells after P + C treatment (Figures 7(g)–7(i)), whereas fewer dead cells were observed in the sample treated with C + P (Figures 7(d)–7(f)). The round black region at the center in Figure 7 also appears to enlarge with increasing treatment time, highlighting the dose dependency of plasma treatment. Also, the 3D images in Figures 7(c), 7(f), and 7(i) show a clear picture of the treated biofilm with a black spot visible at the center with Figures 7(b), 7(e), and 7(h) consisting of a single image taken at the treated region. Also, Figures 7(a), 7(d), and 7(g) comprise multiple images stitched together to get the bigger image of the treated and control biofilm on the titanium disc.

## 5. Discussion

Cold atmospheric plasma combined with other antimicrobial agents could serve as a novel method for the destruction of biofilms on inanimate surfaces as shown in this study. In developing such treatment, using a biofilm model that mimics biofilms grown* in vivo* with the related nutrient sources and substratum is critically essential. Hence, in this study, we used a biofilm reactor to grow biofilms for 48 hours, which resemble the biofilm grown* in vivo. *We then administered treatment with plasma, the disinfectant CHX, and a combination of both treatments in different orders. Significantly, these biofilms were grown on a titanium surface, which is a material often contaminated with biofilms in the hospital environment [41]. Additionally, we choose* P. aeruginosa* as the bacterial species with which we can test our biofilm treatment, as it is a deadly and common pathogen present in the hospital environment and is difficult to eradicate.

In this study, we noted a significant difference in the reduction of viable biofilm cells between the two treatment orders: C + P and P + C. Our results are in agreement with another study [24] regarding increasing efficacy when plasma is combined with other disinfectants. Their results showed greater antimicrobial efficacy when the dental biofilm was treated with plasma before biocide treatment (NaOCl and H_2_O_2_). On the other hand, this research group also demonstrated increased antimicrobial efficacy when CHX and other biocides (PHMB, EDTA, and OCT) were applied before plasma treatment. In contrast, our results illustrate more bacterial killing when plasma was used prior to CHX than with CHX applied prior to plasma. We established complete sterilization (no colonies detected) of the titanium surface when plasma was applied prior to CHX within 5 minutes and a >4 log reduction in bacterial numbers when CHX was applied prior to plasma within 15 minutes of treatment (Figure 4), which is different from the study mentioned above. Therefore, treating biofilms with plasma prior to CHX treatment is a highly efficient method for killing bacteria in biofilms.

The exact mechanism by which the bacteria in biofilms are killed when plasma was applied prior to CHX (P + C) is not entirely understood. One possible reason for these results might be the synergistic interplay between plasma and CHX application in this two-step treatment. First, when plasma is applied, various plasma-produced reactive species penetrate the ECM and disrupt it. The disruption happens as the reactive species, passing through the outer membranes, interact with the inner cell membrane via lipid peroxidation [42–44]. Although plasma may cause cells to leak and reactive species to interact with the intracellular components causing further cell damage, it should be noted that plasma treatment itself did not result in complete sterilization of the biofilm. In the second step of treatment, when CHX is applied, the biofilm is already disrupted and partially inactivated. Upon application of CHX, further cell leakage occurs and CHX interacts with the remaining intracellular components that were left undamaged by the plasma reactive species, resulting in cell death [24, 45], and eventually complete biofilm sterilization is achieved. The improved result could be potentially due to CHX, even at lower concentrations, getting easy access to the intracellular components via the plasma-disrupted ECM. Hence, the physiologically safer low CHX concentration that would normally have only bacteriostatic effect is now able to completely sterilize the biofilm after a previous brief exposure to nonthermal plasma. On the other hand, the results from the C + P treatment were significantly different from the results of P + C treatment. Although it is well known that CHX binds to the cell wall and causes leakage of intracellular components, it might not have been able to disrupt the ECM as effectively as plasma reactive species do. The difference in degree of disruption by both treatments, when applied individually, was observed in SEM images, where CHX treatment produces biofilm clusters but plasma treatment results in highly scattered biofilm cells. Hence, further application of plasma after CHX treatment does not have a complete sterilization effect.

Very few studies examining the effectiveness of plasma and CHX were previously performed [24, 46, 47]. Du et al. and Herbst et al. worked with dental biofilm and found a significant reduction of biofilm cells when treated with CHX first and plasma later. However, they did not investigate treatment with plasma first and CHX later as was done in our present study. In addition, in this study, the biofilms were grown in a reactor with flow for 48 hours aiming to simulate the natural biofilms that can be formed in our body for the first time to our knowledge.

Along with the colony count assay, an XTT assay was employed as this assay has frequently been used for quantifying biofilms because it detects the presence of cells that are viable and metabolically active [23, 48, 49]. The low absorbance reading in P + C treatment signifies the absence of metabolically active cells in comparison to the NaCl control. Moreover, the absorbance reading of P + C is similar to the blank control, which further validates the absence of viable bacterial cells on the titanium surface (Figure 5). The results from both the colony count and XTT assays demonstrate the efficient killing of biofilms with our combined treatment approach.

SEM was used to observe the efficacy of the combination treatment method on biofilms. The biofilm with negative control NaCl does not have any physically disrupting effect on biofilm as seen on Figure 6 as the biofilms are intact and clustered together. The individual treatments using plasma or CHX alone result in more biofilm destruction as compared to the NaCl control. The level of degradation and destruction of biofilms in our treated sample (C + P and P + C) is similar to another study [50–52], where the biofilm degradation was achieved using the biocides NaOCl and CHX. In a study performed by Lunov et al. [53], the destruction of biofilms is similar to what we observed for P + C treatment, where the group used nonthermal plasma (He jet) on Gram-negative and Gram-positive bacteria. This group used helium plasma to destroy bacteria and demonstrate that the plasma treatment is safe to use for MSC (mesenchymal stem cells) and skin cells, mainly highlighting plasma applications for chronic wound therapy. Thus, the presence of no surviving bacteria after the P + C treatment in our study suggests the potential of this method for future clinical applications.

In the confocal images presented here, more yellow or dead biofilm cells were observed in plasma treated samples in comparison to CHX treated samples (Figure 7), which signifies greater killing of bacteria by plasma than by CHX. The images also demonstrate that treatment with plasma first is more effective than treatment with CHX first when the two treatments are used in combination. The negative control (NaCl treated) biofilm shows green fluorescence (live bacteria), and the intensity of the yellow color (dead) is higher for P + C treatment than it is for C + P treatment. This suggests that the majority of the cells in the P + C treated biofilm are dead. Also, the maximum biofilm thickness observed by the confocal microscope in NaCl and CHX control was between 40 um and 60 um. However, the thickness of the other treated samples at the treated area was hard to measure because the center treated region (black spot) has zero thickness or depth.

More studies need to be performed in this new research area to determine the underlying mechanism responsible for killing bacteria in biofilms using this combination treatment methodology. Additional work is needed to investigate how applying plasma prior to CHX sterilizes biofilms more efficiently than applying CHX before plasma.

## 6. Conclusion

This study shows that increased killing of biofilms can be obtained when a combination of treatments is used over using any individual treatment by itself. Treatment with plasma prior to CHX treatment resulted in complete sterilization of a biofilm-contaminated titanium surface. Interestingly, >4 log reduction in bacterial cell numbers was observed when CHX treatment preceded plasma treatment. This combined treatment strategy is advantageous as it would allow for complete sterilization of surfaces with clinically safe doses of plasma and concentrations of CHX. Therefore, we propose that the combination treatment method should be considered as a promising future method for the sterilization of medical devices and biomaterials.

## Figures and Tables

**Table 1:** Effect of inactivation solution on planktonic bacteria and biofilm viability.

Treatment method	Surviving cells/ml on planktonic bacteria	Surviving cells/ml on biofilm
Untreated biofilm	6.50*E* + 07	3.8*E* + 07
Biofilm treated with inactivation solution only	6.50*E* + 07	2.5*E* + 07
